# Research, development, and evaluation of the practical effect of a storage inflow and outflow management system for consumables in the endocrinology department of a hospital

**DOI:** 10.1186/s12911-021-01744-y

**Published:** 2022-01-11

**Authors:** Jiang Luo, Yan Wang, Yongze Zhang, Xiaofang Yan, Xiaoting Huang, Fengying Zhao

**Affiliations:** 1 Department of Endocrinology, The First Affiliated Hospital of Fujian Medical University, 20 Cha Zhong Road, Fuzhou, 350005 Fujian China; 2 Clinical Research Center for Metabolic Diseases of Fujian Province, the First Affiliated Hospital of Fujian Medical University, 20 Cha Zhong Road, Fuzhou, 350005 Fujian China; 3 Diabetes Research Institute of Fujian Province, the First Affiliated Hospital of Fujian Medical University, 20 Cha Zhong Road, Fuzhou, 350005 Fujian China; 4 Metabolic Diseases Research Institute, the First Affiliated Hospital of Fujian Medical University, 20 Cha Zhong Road, Fuzhou, 350005 Fujian China

**Keywords:** Consumable storage inflow and outflow management system, Consumable management, Information based, Nursing management

## Abstract

**Background:**

This study was designed for the research and development (R&D) and application of a storage inflow and outflow management system enabling departments to perform efficient, scientific, and information-based consumable management.

**Methods:**

In the endocrinology department of a hospital, expert and R&D teams in consumable management were set up, and an information-based storage inflow and outflow management system for consumables was designed and developed. The system was operated on a personal computer and was divided into three modules: public consumables, bed consumables, and quality control management. The functions of the system included storage inflow and outflow, early warnings, response to user queries, and statistics on consumables. Data were derived from the hospital information system (HIS,ZHIY SOFTWARE HIS VERSION4.0) and a questionnaire survey. Economic indicators, work efficiency of consumable management, nurse burnout, consumable stockroom management, and staff satisfaction were compared under manual management, Excel-based management, and the consumable storage inflow and outflow management system. The results of the questionnaire were analysed using the R software, version 4.1.0.

**Results:**

Dates were obtained from manual management, Excel-based management and the consumable storage inflow and outflow management system. Under these three methods, the daily prices of department consumables per bed were 53.43 ± 10.27 yuan, 38.65 ± 8.56 yuan, and 31.98 ± 7.36 yuan, respectively, indicating that the new management system reduced costs for the department. The time spent daily on consumable management was shortened from 119.5 (106.75, 123.5) min to 56.5 (48.5, 60.75) to 20 (17.25, 24.25) min. Nurses’ emotional fatigue and job indifference scores, respectively, decreased from 22.90 ± 1.65 and 8.75 ± 1.25 under manual management to 19.70 ± 1.72 and 6.90 ± 1.37 under Excel-based management and to 17.20 ± 2.04 and 6.00 ± 1.30 under the novel system; the satisfaction of the warehouse keeper and collection staff, respectively, increased from 76.62% and 80.78% to 91.6% and 90.5% to 98.8% and 98.5% under the three successive systems.

**Conclusions:**

The storage inflow and outflow management system achieved produced good results in the storage and classification of consumables.

**Supplementary Information:**

The online version contains supplementary material available at 10.1186/s12911-021-01744-y.

## Background

High numbers of consumable medical materials (e.g., sterile needles and swabs) are used by hospital medical departments worldwide every day. Medical consumable management is an important part of medical care management. Many hospital departments need to track the individual and overall use of materials. The effectiveness of management has an observable impact on the operating costs and productivity of a department [1, 2]. In June 2019, the National Health Commission of China and the National Administration of Traditional Chinese Medicine categorically declared that medical consumable management at medical institutions should be strengthened to promote the reasonable use of these resources.

Medical consumables can be divided into high-value and low-value consumables based on their pricing, and they can be classified as chargeable or nonchargeable consumables based on whether they are free for patients or come at a cost. For high-value operating room consumables [3], zero inventory management involving code scanning and charging is used [4, 5], which can make the quantities of collection and use of consumables reach 100%. Supply management of medical consumption materials is based on the supply, processing, and distribution (SPD) mode, and consumable inventory management is information based [1, 6, 7]. Post et al. [8] proposed the construction of the information-based data warehouse management system of the i2b2 Data Warehouse. Davoody et al. [9] proposed the construction of a hospital information exchange platform and cost control management for noncharging consumables. However, few studies have described the actual overall scheme and details of the design and operation of consumable management for specific departments. It is important to investigate the monthly consumable collection and the quantities of monthly use of different consumable medical materials. Effectively controlling the inflow**–**outflow balance of consumables is important in optimizing the operation of a department.

It is imperative to refine the management of medical consumables according to various health-care policies enacted in recent years. Implementing scientific and rational management of medical consumables is one way for a hospital to keep pace with new policies. At present, the prevalence of diabetes is high [10], and many outpatients and inpatients in hospital endocrinology departments have diabetes-related complications [11]. The increase in complications has raised the demand for clinical medical consumables. In this study, the consumable management systems implemented by the endocrinology department of The First Affiliated Hospital of Fujian Medical University since 2013, from manual management to management based on Microsoft Excel tabulation and then to the storage inflow and outflow management system, are described and evaluated.

## Methods

### Overall design

Since 2013, the department has been exploring the effect of consumable management, including management based on Microsoft Excel tabulation and the later application of failure mode and effect analysis (FMEA) combined with root cause analysis (RCA) [12]. Additionally, an in**–**out storage management system for the endocrinology department was developed and used.

### Management mode

#### Manual management mode

Consumable application forms were collected and evaluated monthly. Consumables that were not charged as per the doctor’s advice were registered in a paper book after they were used, and those that were charged were recorded in the hospital information system (HIS) by a specially assigned person. After an account was made of all the consumables used at the end of each month, the departmental collection data and the data from the HIS were compared with the manual paper registration data to obtain statistics on consumable management in the department.

#### Excel-based management mode

Consumables were divided into two Excel spreadsheets, chargeable and nonchargeable. Each consumable item was accounted for using the following four headings: original inventory, new stock, used inventory, and remaining inventory. On the first day of each month, the remaining inventory of the previous month was entered as the original inventory, the supplies acquired in the current month were the new stock, the supplies consumed by routine use were entered as the used inventory, and the remaining inventory was automatically calculated and displayed in real time (original inventory + new collection – used inventory). At the end of each month, the departmental collection data and the data from the HIS were compared with the data in the Excel sheets to obtain statistics on consumable management in the department.

#### Consumable storage inflow and outflow management system

Expert and R&D teams were established for consumable management. The expert team consisted of eight persons, comprising one medical nursing expert, one surgical nursing expert, two supervisor nurses working on the clinical frontlines, two information experts, and two consumable management experts, who were responsible for reviewing and forming the first draft of the system design. After three rounds of expert team meetings, improvements were made in the aspects of scientific rigor, practicability, safety, and convenience, and a draft was ultimately handed over to the R&D personnel for development, testing, and online use. The R&D team consisted of 10 persons, comprising 2 head nurses, 2 senior nurses, 2 clinical nurses with intermediate professional titles, 2 information engineers, and 2 consumable management engineers, who were responsible for development, testing, adjustment, and application guidance.

**Fig. 1:**
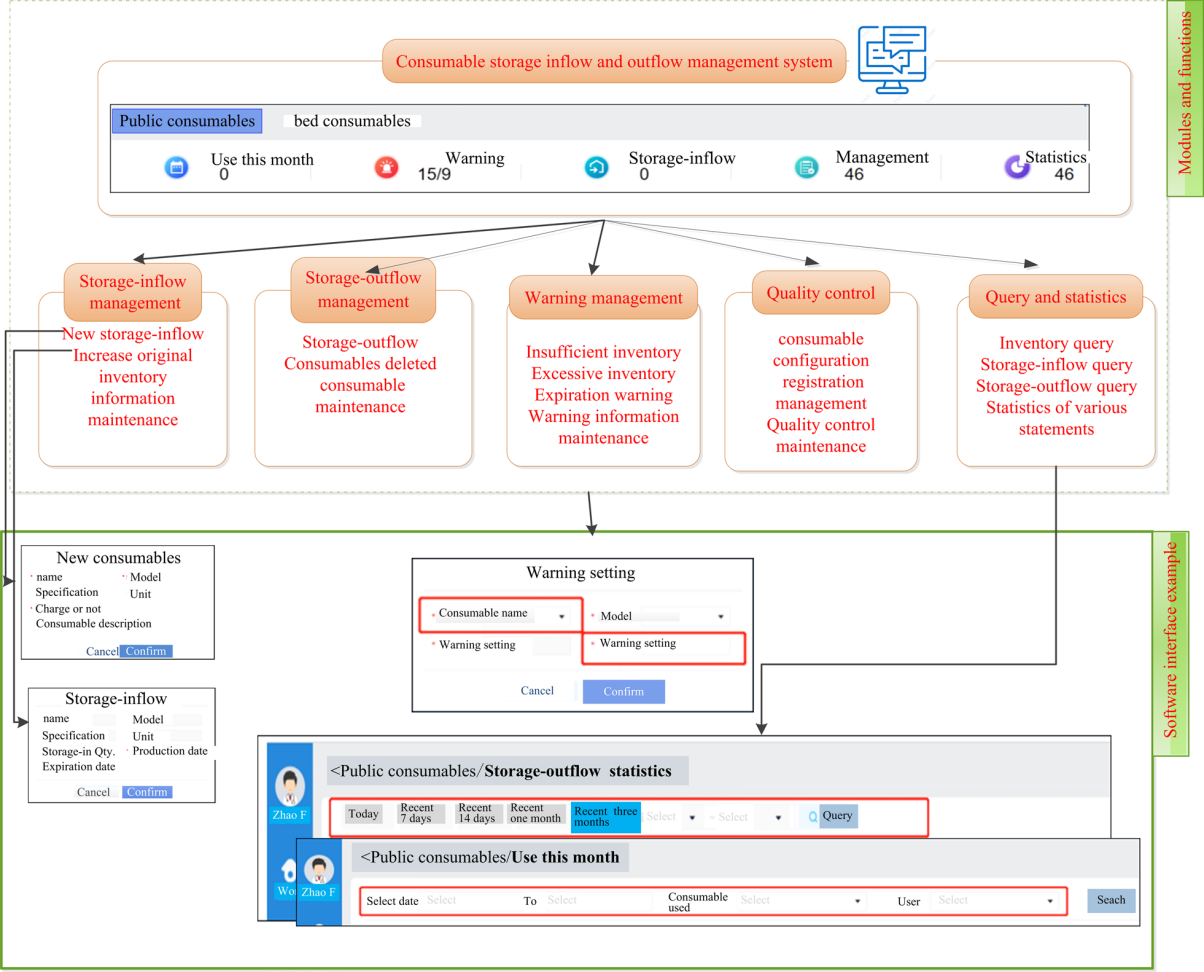
Introduction to the module functions and software interfaces of the in**–**out storage management system. The picture is a translated excerpt from the in**–**out storage management system for consumables described in this paper. Reference the attached software copyright certificate for details

The storage inflow and outflow management system was connected to the supply room, equipment warehouse, administrative warehouse, and HIS. The inventory increased automatically after the consumable application form was issued, and the consumable was automatically listed as part of the storage outflow after the consumer was charged. Nonchargeable consumables were released from storage in association with the doctor’s advice or manual entry. The management system was divided into three parts: public consumables, bed consumables, and quality control management. The specific modules, an introduction to the functions, and a demonstration of the software interfaces are shown in Fig. 1.

**Fig. 2:**
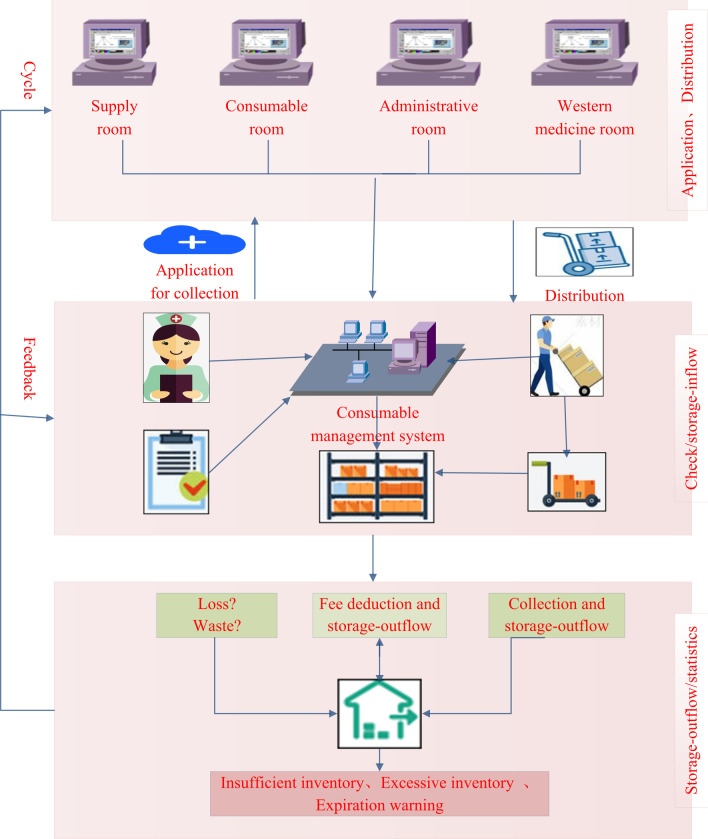
Closed-loop management of consumables. The picture is a translated excerpt from the in**–**out storage management system for consumables described in this paper. Reference the attached software copyright certificate for details

Through the management system, the head nurses and the consumable management team made a statistical comparison of the quantities of storage inflow and outflow, the quantity charged according to the HIS, the quantity of remaining inventory as recorded in the system, and the actual inventory of the department. Key consumables were tracked every week. If these values were approximately consistent, the collection, use, and charge matching, as well as the department’s consumable management, were found to be reasonable. If the difference was significant, the department faced problems such as possible overstocking of consumables, serious loss, repeated charging, charge missing, and loss of consumables, among others. In the case of an inconsistency, FMEA, RCA, and brainstorming were applied to determine the causes, rectify the problem, and adjust the collected amount in a timely manner [13]. The inventory consumables were checked regularly to ensure that the accounts were consistent with the goods and with each other by real-time monitoring and evaluation through the consumable management system. The closed-loop consumable management system comprised application submissions, distribution and checking, storage inflow and outflow, statistics/feedback, and evaluation/adjustment of applied quantities and types. A brief flow chart is shown in Fig. 2.

### Data source

The consumable management practices of the endocrinology department were taken as the research object. Data were collected from three periods during which different modes of management were used: manual record management from January 2013 to February 2015 (hereinafter referred to as the manual management group, Group 1), Excel computer software from March 2015 to February 2018 (hereinafter referred to as the Excel management group, Group 2), and the application management system from March 2018 to December 2019 (hereinafter referred to as the management system group, Group 3). The names and specifications of all low-value consumables were constant and thus comparable across these three periods.

### Evaluation indicators

Daily consumable use per bed, also called daily cost per bed, was calculated as follows: daily consumables per bed = [direct cost to the department (total consumable cost for items under 1000 yuan) and indirect cost (water and electricity cost)]/[number of beds × total days (days per month)].

The quantity of monthly consumable storage outflow is the total cost of the consumables under 1000 yuan that are used by the department in one month.

A missed bookkeeping event refers to a failure to charge for an item due to a failure to register, record, or statistically analyse the transaction on time after the use of the consumable.

Expired consumables are defined as medical consumables that are beyond their expiry date due to overstocking, going unnoticed, low frequency of use, or noncompliance with the collection specifications.

Daily inventory time refers to the time required by nurses for daily registration and checking of medical consumables as part of consumable management.

Time spent by main shift nurses on discharge settlement, measured in person-time, is the time it takes the nurse to check the use of medical materials and the corresponding charges when a patient is discharged.

In the blood glucose monitoring management system, a portable blood glucose metre was connected to the hospital HIS system, and the blood glucose results were matched one to one with the patients’ information. The system automatically generated each patient’s fixed-point blood glucose recording table and trend chart and displayed the number of blood glucose measurements from the patient in real time. The number of blood glucose results was equal to the number of blood glucose test strips consumed.

The monthly consumable collection frequency is the number of times medical consumables are requested per month.

The match between the quantities of monthly collection and use was calculated as follows: match = [amount collected/amount used]*100%.

A job burnout scale [14] for nurses was used to interpret the questionnaire data from the Maslach Burnout Inventory. The inventory had 22 items spanning 3 dimensions: emotional fatigue, job indifference, and lack of a sense of achievement. Each item was scored from 0 to 6 points, and the scores were summed within each dimension. The emotional fatigue dimension included nine positively scored items that evaluated emotional reaction caused by work pressure, for a total score of 0–54 points. The dimension of job indifference included five items, which evaluated objectifying attitudes and feelings towards patients due to work pressure; these items were scored positively, and possible scores on this dimension ranged from 0 to 30. The dimension of lack of a sense of achievement included eight items. It evaluated this effect of work pressure on a reverse scale, with a possible total score of 0–48. The scale was demonstrated to have good internal consistency. For the dimensions of fatigue, job indifference, and lack of a sense of achievement, Cronbach’s alpha coefficient (a measure of reliability) was 0.837, 0.869, and 0.881, respectively. The survey was administered to all 22 nurses in the endocrinology department.

#### Satisfaction of warehouse keeper and collection staff

Satisfaction is most commonly assessed with a single-item instrument such as the visual analogue scale (VAS). The satisfaction VAS is a 10-cm straight line with labels on each end to anchor the scale from ‘not satisfied at all’ to ‘completely satisfied’ [15, 16]. A satisfaction questionnaire (Additional file 1) that included the time of collection, the duration of collection, and the accuracy of nurses’ filling in the form, planning of receiving, times of replacement, dedicated management, reasonable process, time consumption, response speed in case of abnormality, and overall evaluation. There were 10 items worth 10 points each, for a total of 100 points. Staff were asked to place a moving marker on the line to represent their satisfaction, and the position of the marker was measured in millimetres. The distance marked along the line corresponded to a satisfaction score from 0 to 10. The survey questionnaire, with a maximum score of 100 points, was graded in terms of satisfaction: very low (under or equal to 30 points), low (31 to 50 points), moderate (51 to 80 points), high (81 to 92 points), and very high (93 to 100 points). The data were analysed using the R software, version 4.1.0 All data were presented as the mean and SD. For internal reliability, Cronbach’s α was 0.859 (values ≥ 0.7 for the overall score and for each section were considered adequate) [17]; for external reliability, a paired t-test was performed, and an intra-class correlation coefficient of 0.844 was calculated (intra-class correlation coefficient values ≥ 0.7 were considered adequate). Excluding temporary personnel, a total of 5 warehouse keepers and 4 collection staff members participated in the investigation. The questionnaire was self-administered to safeguard the anonymity of the study participants.

### Statistical analysis

Statistical analyses were performed using the R software, version 4.1.0. Graphs were drawn using Adobe lllustrator version 2010. Excel was using WPS Office,version 11.0. Normally distributed measurement data are expressed as the mean ± standard deviation (SD), and abnormally distributed measurement data are expressed as the median (upper and lower quartiles). The significance of differences among groups was determined by a paired t-test and McNemar's test. Comparisons between the job burnout scores of nurses within each group were performed by repeated-measures analysis of variance. A *P* value < 0.05 was considered statistically significant.

## Results

### Effect analysis

#### Quantity of daily consumables per bed and monthly consumable storage outflow

**Fig. 3:**
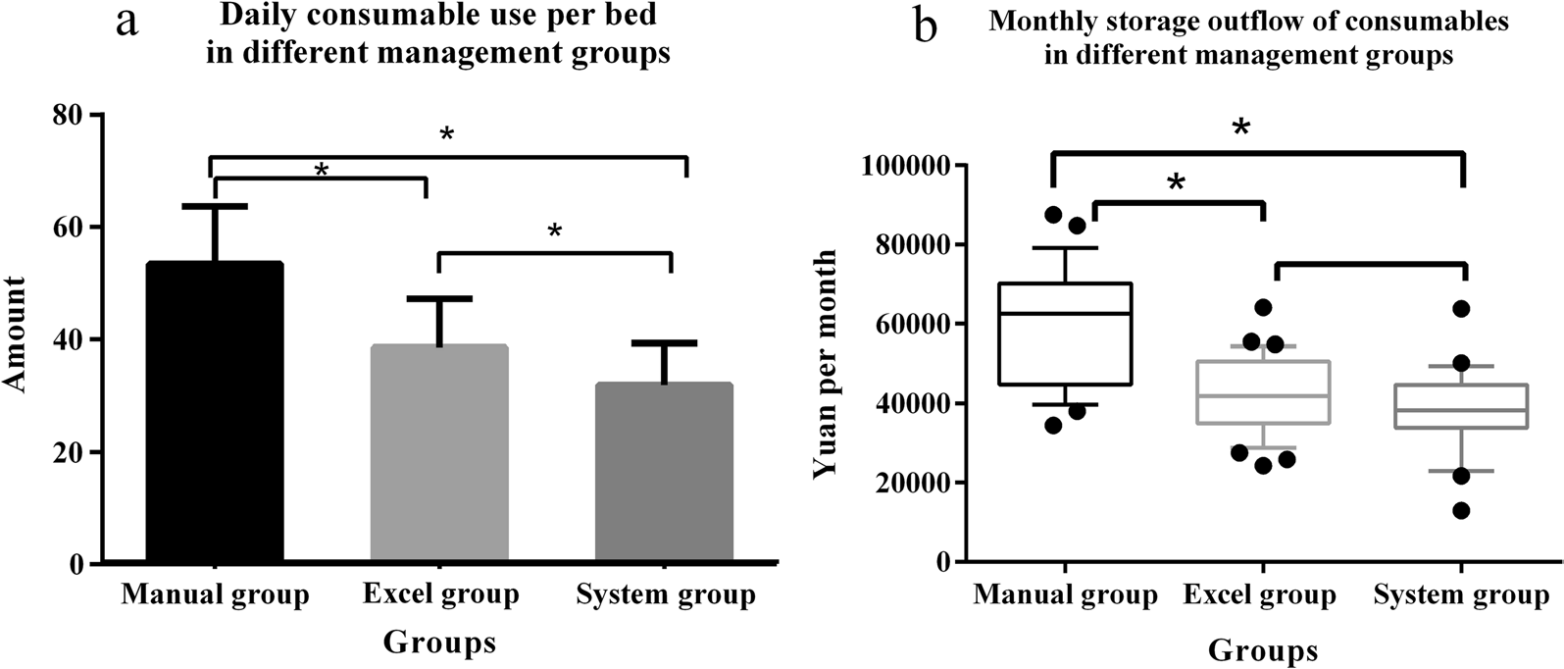
Comparisons of the quantity of daily consumables per bed and monthly consumable storage outflow (^*^P < 0.05). **a** One-way ANOVA and least significant difference for post-test comparisons. **b** Kruskal–Wallis test and Nemeyi method for post-test comparisons

The quantity of daily consumables per bed and the monthly consumable storage outflow of the department were collected from the HIS of the hospital. In Group 3, the cost of daily consumables per bed was 31.98 ± 7.36 yuan, which was 40.15% lower than the cost in Group 1 (53.43 ± 10.27) and 17.2% lower than that in Group 2 (38.65 ± 8.56). The differences between the three groups were statistically significant (*P* < 0.01). The details are shown in Fig. 3.

The monthly consumable storage outflow quantity of Group 3 was 41,907.76 (34,916.91–50,641.29) yuan, which was lower (26.86%) than that of Group 1 (62,523.27 (44,775.68–70,205.81) yuan) by a statistically significant margin (*P* < 0.01); however, no statistically significant difference was noted between Group 3 and Group 2 (Group 2, 39,085.42 (34,878.03–46,022.78) yuan; *P* > 0.05). The comparison of the monthly consumable storage outflow quantities of the three groups (*P* < 0.01) revealed a statistically significant difference, as shown in Fig. 3.

#### Missed bookkeeping and expired consumables

**Fig. 4:**
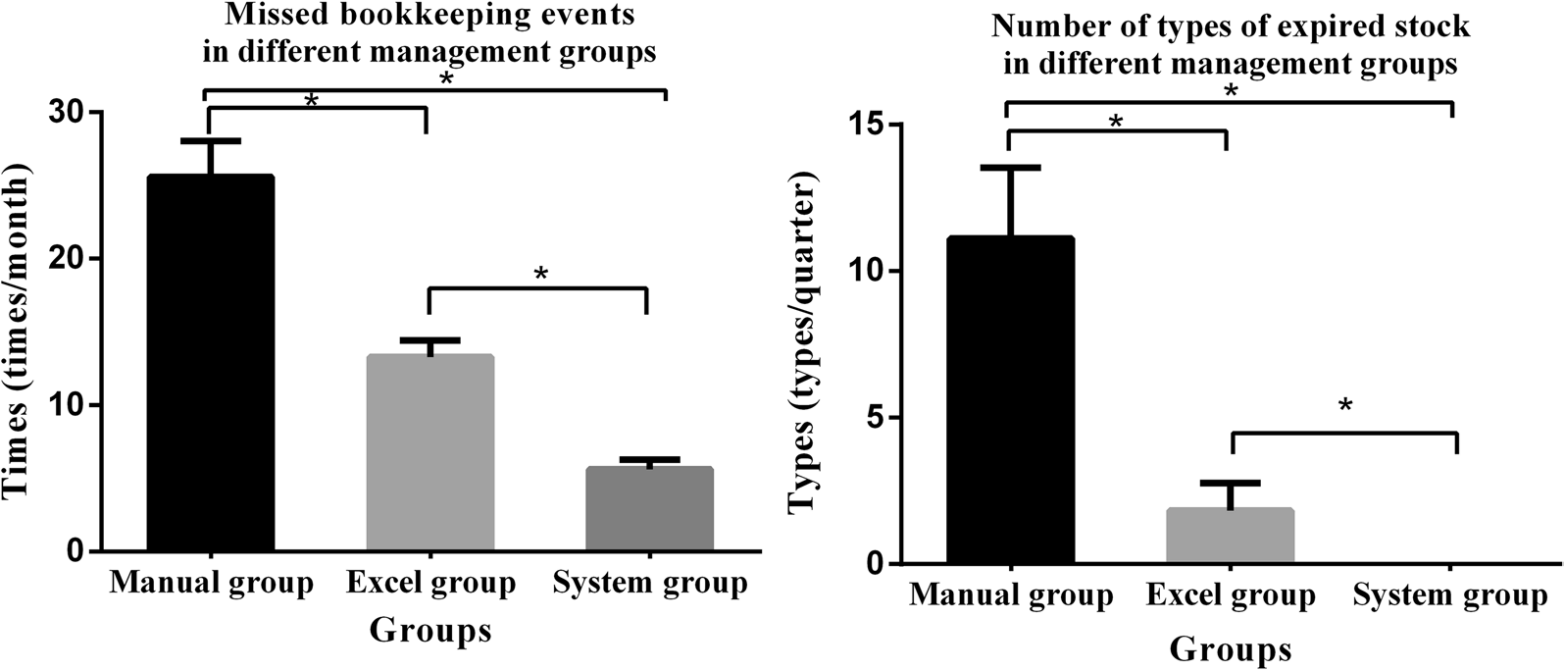
Comparisons of the number of missed bookkeeping events per month and types of expired stock (*P < 0.05). *P* < 0.05 one-way ANOVA and least significant difference for post-test comparisons

The average number of missed bookkeeping events decreased from 26 in Group 1 to 13 in Group 2 and 6 in Group 3, for an overall decrease of 77%. The error rate decreased from Group 1 to Groups 2 and 3, and the difference was statistically significant (*P* < 0.05). The number of missed bookkeeping events in each group is shown in Fig. 4.

The number of expired consumables per quarter decreased from 11.11 ± 2.42 in Group 1 to 1.83 ± 0.94 in Group 2. After the management system was started, no expired consumables were found, which constituted a statistically significant difference (*P* < 0.05). The details are given in Fig. 4.

#### Daily inventory time

**Fig. 5:**
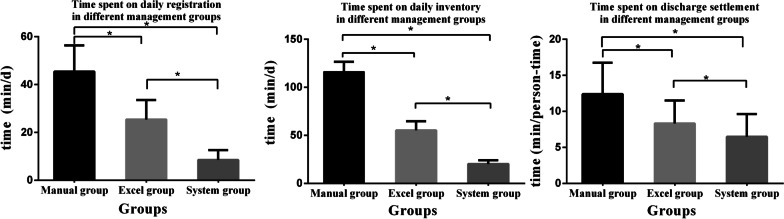
Time spent on registration, inventory, and discharge settlement in different management groups (*P < 0.05). *P* < 0.05 one-way ANOVA and least significant difference for post-test comparisons

The consumable management nurses recorded the time they spent daily registering and checking the inventory, and the main shift nurses recorded the amount of person-time spent on discharge settlement. Data from the same month in three different years, one in each of the three periods, were selected for statistical analysis; these data included daily inventory time, daily time spent by management nurses on registration and checking, and person-time spent by main shift nurses on discharge settlement. The three groups were significantly different in these respects (*P* < 0.05), as shown in Fig. 5.

Consumable overstocking and unrecorded inventory sometimes occur in traditional consumable management. With the consumable management system, the consumables of the whole department were included in management and placed at a fixed point. During the daily/monthly inventory, the remaining inventory according to the system was compared with the actual inventory of the department to ensure consistency of accounts and consistency between the accounts and the actual stock by effective counting, reasonable judgement, accurate tracking, and confirmation by signature after counting. The consistency was as high as 95%–100%. From Group 1 to Groups 2 and 3, the daily inventory time decreased from 119.5 (106.75–123.5) min to 56.5 (48.5–60.75) min and 20 (17.25, 24.25) min, respectively, decreases of 52.72% and 83.26%. Nurses’ daily consumable registration time decreased significantly from 46.00 (39.00–54.00) min to 26.50 (19.25, 32.15) min and 8.00 (4.75–12.00) min (*P* < 0.05), with a maximum decrease of 82.60%, which saved a substantial quantity of nursing resources and increased the time available for the nurses’ clinical duties.

Since 2018, an in-hospital blood glucose monitoring management system has been applied. After the blood glucose monitoring results were saved, they were associated with the patients’ information. The system automatically generated a fixed-point blood glucose recording table of the patient. The monitoring times were quite clear, which saved counting time when settling accounts, avoided the omission or duplication of bookkeeping entries, facilitated the counting of losses, saved the nurses a considerable amount of time checking inventory, improved the work efficiency of nurses during discharge settlement, and shortened the waiting time of patients. The average time spent by main shift nurses on discharge settlement decreased from 16.87 ± 4.48 to 10.97 ± 3.74 and 7.47 ± 2.85 person-minutes. The core reconciliation work was completed efficiently, and the key steps of consumable management were well controlled. The difference was statistically significant (*P* < 0.05).

#### Monthly consumable collection frequency

From manual management to Excel software management and then to the application management system, consumable collection templates with fixed custom base numbers were generated for the department, including a “monthly template for the department and a weekly template for the supply room. The monthly template of the department was reviewed at the beginning of each month to check the number of applications and make appropriate adjustments before the application request was sent out. The weekly template of the supply room was reviewed every Monday to make appropriate adjustments according to the balance of the previous week and to check the quantity before submission. Under the supervision of the head nurse and the specially appointed person in charge, taking the storage space of the department into account, the monthly collection frequency was fixed in what was labelled “1 + 4” mode; that is, 1 × the expected demand for common consumables and 4 × the expected demand for supply room consumables were stocked. Although consumable safety management was conducted properly, a reasonable reserve of consumables was ensured, and waste was eliminated. The number of monthly collection applications decreased from 40–50 to approximately 10, a decrease of at least 75%. Thus, the expenditure of time and labour on collection was streamlined.

#### Types and quantities of consumables under departmental management

After the implementation of consumable management, the number of types of consumables under department management decreased from 286 types (with an average of 32,952 items per month) to 133 types (with an average of 22,265 items per month). The variety and quantity of consumables were reduced.

#### Matching the quantities of monthly collection and use to form an intelligent storage and use model

**Fig. 6:**
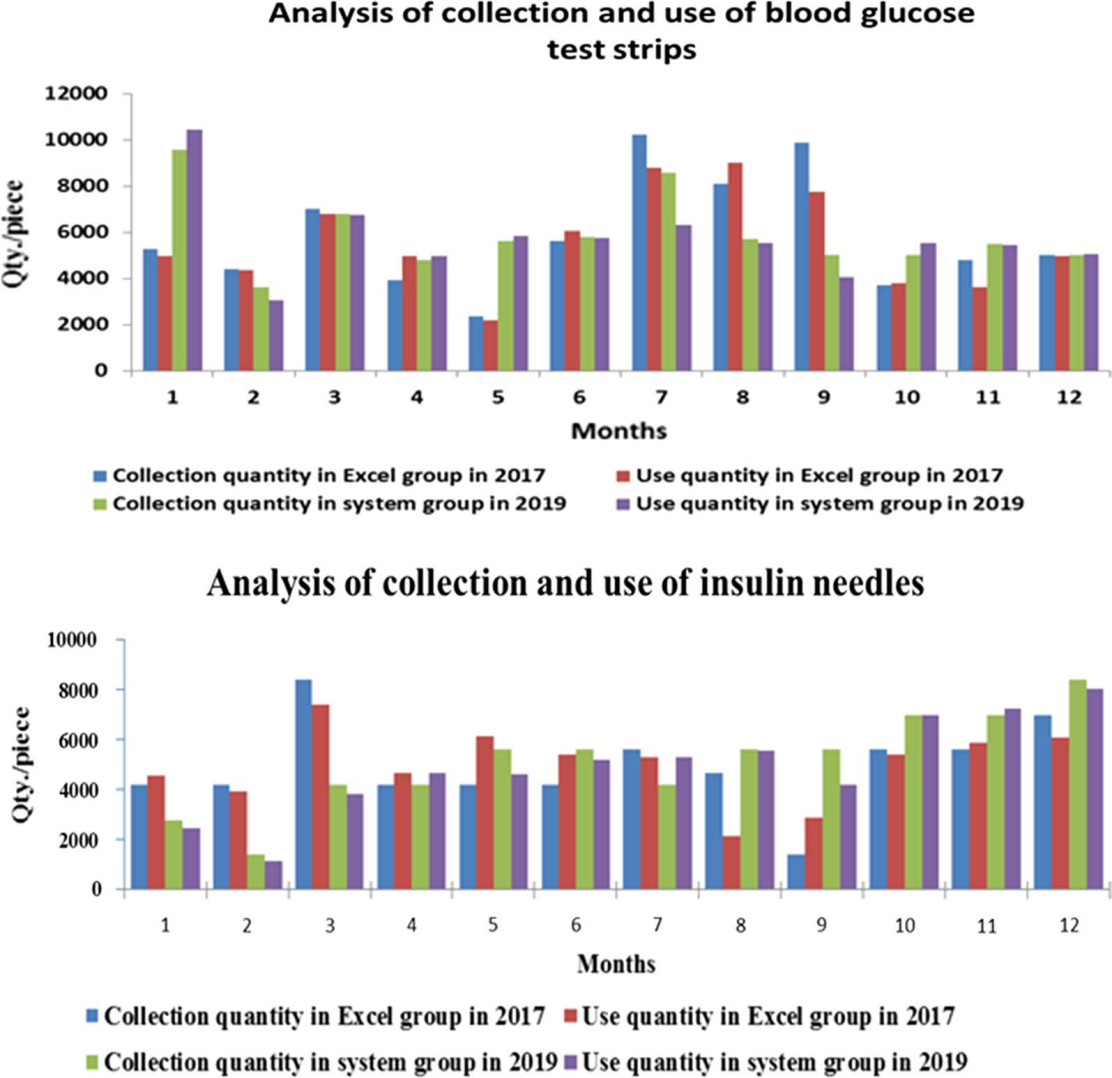
The collection and use of blood glucose test strips and insulin needles in different groups

As suggested by the retrospective statistical analysis, the match between the collected and used quantities of the two most commonly used special consumables, that is, blood glucose test strips and insulin injection needles, fluctuated between 95 and 105%, on average, which was within the safe margin for the inventory [18, 19]. Figure 6 shows the quantities of blood glucose test strips and insulin injection needles collected and an analysis of their use in Group 2 in 2017 and in Group 3 in 2019. The average ratio of collected to used blood glucose test strips in 2017 and 2019 was 1.0452 ± 0.154 and 1.0472 ± 0.138, respectively. The *P* value for comparison among the groups was 0.974, indicating no statistically significant difference. The average collection/use ratio of insulin needles in 2017 and 2019 was 1.0276 ± 0.410 and 0.954 ± 0.140, respectively (*P* = 0.562), showing no statistically significant difference. However, the standard deviation of insulin needle matching was lower in 2019 than in 2017; thus, collection became a more precise predictor of use. As shown in Fig. 6, the quantity of blood glucose test strips collected exceeded the quantity used in September because the National Day holiday was observed from October 1 to October 7; in anticipation, additional materials were collected in September to ensure a sufficient supply during the holiday. Since insulin needles were collected in boxes (1400 pieces), the collection of insulin needles in Group 3 was steadier than that of glucose test strips under the system.

#### Nurse burnout

The endocrinology department had 22 nurses aged 33.72 ± 7.93 years. The emotional fatigue scores of Group 2 and Group 3 were 19.70 ± 1.72 and 17.20 ± 2.04, and the job indifference scores were 6.90 ± 1.37 and 6.00 ± 1.30, respectively. These values were lower than those of Group 1 (22.90 ± 1.65 and 8.75 ± 1.25, respectively). These differences were statistically significant (*P* < 0.05). Specific results are shown in Table 1.

**Table 1:** Comparison of burnout scores of nurses in different management groups (*n* = 22; *P < 0.05)

	January 2013 to February 2015	March 2015 to February 2018	March 2018 to December 2019	*p*
	Manual group	Excel group	System group	
Emotional fatigue	22.9 ± 1.65	19.7 ± 1.72^a^	17.2 ± 2.04 ^ab^	< 0.001
Job indifference	8.75 ± 1.25	6.9 ± 1.37 ^a^	6 ± 1.3 ^ab^	< 0.001
Lack of sense of achievement	35 ± 4.4	40.65 ± 3.12 ^a^	42.1 ± 2.61 ^a^	< 0.001

^a^Comparison with the manual group *p* < 0.05, ^b^Comparison with the Excel group *p* < 0.05;

*P* < 0.05, Repetitive measure analysis of variance (ANOVA) and least significant difference for post-test comparisons

After the implementation of consumable management, the responsible post and the night-shift post were established to collect consumables needed by patients during the shift and register them after use (no registration was required for automatically associated consumables) without additional responsibilities, such as the collection of supply room consumables, helping nurses achieve better mood and higher work satisfaction as they devoted themselves to clinical nursing. The scores for lacking a sense of achievement in Group 2 (40.65 ± 3.12) and Group 3 (42.10 ± 2.6) were higher than those in Group 1 (35.00 ± 4.40), indicating that nurses had greater professional identity and job pleasure. The differences in nurses’ job burnout scores under different methods of consumable management were statistically significant (*P* < 0.05). Specific results are shown in Table 1

#### Satisfaction of warehouse keeper and collection staff

An in-house questionnaire, which included the times of collection, the duration of collection, and the accuracy with which nurses filled out the form, was adopted. After the “1 + 4” collection mode was implemented, the collection times were relatively fixed. The collection workers and warehouse keepers of the endocrinology department developed corresponding work habits, which shortened the waiting time of nurses for consumables. The satisfaction of warehouse keepers and collection staff improved from 76.62% and 80.78% to 91.6% and 90.5% to 98.8% and 98.5%, respectively, over the three study periods.

## Discussion

With the deepening of national health-care reform, the management requirements for medical consumables were raised to a new height: in 2019, the Management Measures for Medical Consumables of Medical Institutions formulated by the National Health Commission required that a full process of information-based management of the supply, use, supervision, and analysis of medical consumables be implemented and that every medical consumable be tracked throughout its life cycle. With the growth of the health-care sector, the proportion of medical consumable costs as a share of overall medical costs has gradually increased [20, 21]. When a zero price difference was implemented, the effect of management was decisive in ensuring the efficient and normal operation of hospitals and departments.

According to the use and management experience of clinical departments, hospital consumables were divided into three categories: high value, low value, and nonchargeable. Additionally, all consumables were divided into two categories according to their destination: patient use and public use. In the 2004 edition of the Medical Service Prices of Provincial Medical Institutions in Fujian Province issued by the Fujian Provincial Price Bureau and Fujian Provincial Health Commission, relevant items to be charged for clinical services were clearly defined. For example, the gauze, bandage, dressing bag, and gloves for clinical debridement and dressing were not to be charged additionally. At present, the scope of nonchargeable medical consumables is gradually expanding and now includes most blood-collecting containers for venous blood collection. Medical record forms, pens, masks, hats, iodophors, ethyl alcohol, and other nonchargeable materials commonly used in the department are also subject to price increases and are essential consumables that cannot be cut down. In general, the daily cost of consumables per bed decreased from 53.43 ± 10.27 in Group 1 and 38.65 ± 8.56 yuan/month in Group 2 to 31.98 ± 7.36 yuan/month in Group 3. However, the operating cost of the department still increased to 39,085.42 (34,878.03–46,022.78) yuan/month in Group 2 and 41,907.76 (34,916.91–50,641.29) yuan/month in Group 3: there was no significant difference between these two groups. Consumable management by the department was an overarching task that needed to integrate the high-value, low-value, and nonchargeable consumables to ensure the traceability of their use to reduce the operating costs of the department. The consumable management system used in this study included functions for automatic connection and manual entry, and all categories of consumables were commonly used. Some high-value consumables not commonly used by the department were added after use according to the amount charged, which was conducive to the statistical analysis of consumable management.

This innovative study comprehensively introduced the development, functions, practical operation, and effectiveness evaluation of the management system from the perspective of the departmental consumable management staff, users, and participants; the study is contextualized with references and reported with an emphasis on reproducibility. The consumable management system was useful for HIS-based big data analysis, provided professional management services for clinical practice, and enhanced record keeping for the active management of medical consumables and the enforcement of related policies. Through a unified coding system, medical institutions have optimized the traceability management of high-value consumables, strengthened the tracking of low-value consumables throughout the life cycle, and improved the information sharing rate of the whole process [4, 6, 14, 22, 23], thus improving the efficiency of logistics management, streamlining and standardizing the management of medical consumables in institutions [22, 24], and reducing the consumption proportion.

SPD is a logistics management model for hospital departments [1]. The SPD model can improve the efficiency of medical consumable management in clinical nursing departments, promote the cooperation and participation of all parts of the hospital [25], reduce medical risks and disputes, save hospital operating costs, and decrease capital occupation [26]. In a prior study of the SPD model, the average time spent on inventory per week decreased; the average satisfaction score with management increased, reaching 100%; and the average daily cost of medical consumables decreased. The present study reached similar conclusions, such as a decrease in daily consumables per bed: the cost of daily consumables per bed was 31.98 ± 7.36 yuan in Group 3, which was 40.15% lower than the cost in Group 1 (53.43 ± 10.27) and 17.2% lower than the cost in Group 2 (38.65 ± 8.56). The present study also observed an improvement in nurses’ job burnout scores, a reduction in daily inventory time, and a decrease in discharge settlement time per bed.

SPD requires a specialized management information system and high infrastructure investment. Overall, in mainland China, only six hospitals to date have integrated the SPD model into their hospital management systems. The traditional way to claim consumables is to fill out an application form using office automation (OA) systems. Group 1 and Group 2 in this study used this method. However, OA is not a purpose-designed medical supply management software. The storage inflow and outflow management system for consumables (Group 3) in this study is medical consumable management software that connects with the hospital information system; the department can view the information on the inventory of consumables, such as the quantity, production date, properties data, expiry date/warranty date, chargeability, and description of each consumable. This functionality is advanced, practical, and consistent with the new SPD application system. In particular, with the cooperation and participation of the hospital information department, the storage inflow and outflow management system is connected to the existing information system of the hospital to form an internal closed loop. It does not need the support of various logistics systems and procurement systems and can be widely used in small local hospitals, communities and general hospitals. In contrast, the SPD model is limited in this respect.

Although medical consumables contribute greatly to total hospital expenditure, many hospitals do not track the individual use of materials, for example, with barcodes or radio frequency identification [27]; these tracking systems require specialized material preparation and high infrastructure investment. A novel contactless visual recognition system for tracking medical consumables in intensive care units (ICUs) using a deep learning approach on a distributed client–server architecture has achieved the goal of recognizing materials without explicit labelling. It can accurately identify the use of consumables for bed patients and reduce costs associated with consumable materials [5]. The storage inflow and outflow management system in the present work divides consumables into three categories: public consumables, bed consumables, and quality control management. Statistics on the use of public consumables are kept at the level of the department inventory. Bed consumables are those that are used for patients in individual beds. After the consumables are automatically charged according to the doctor's orders or deducted manually by the nurse, the system will automatically retrieve information on the consumables used for each bed. Some materials that are not charged, such as gauze and bandages, are classified as matching consumables for medical orders. The system identifies the consumables used for each bed after the doctor’s orders are entered. The system clearly presents the total use of consumables and the individual use of consumables. Thus, we believe that the system will ultimately enable hospitals to reduce costs associated with consumable management and consequently let nurses spend more time providing higher-quality care.

The traditional consumable management method is characterized by many disadvantages, such as a low degree of informatization, flaws in the process, a need for extensive management, and consumption of clinical manpower. The size of the nurse workforce has long been insufficient [28]. The consumable management system in this study is an effective way to address the human resources aspect of medical consumable management. Consumables are applied in all forms of medical care. If there is an increase or no change in human resources, consumable management can be added to the nursing responsibilities of each shift. The consumable management responsibilities of each shift are clear and are subject to the overall control of the management system to ensure the orderly development of consumable management. The management system can help department managers quickly and accurately assess the inventory of all varieties of medical consumables, apply this information, and then stock consumables in a timely manner according to the needs of the department to reduce inventory-related burdens on nurses and improve their work efficiency [29]. The consumable management roles of each position are clear, the match between consumable use and charging is improved, the difficulty and complexity of the responsibilities of each shift are reduced, and nurses’ participation in consumable management becomes more enthusiastic. With the implementation of the consumable management system, the daily use of consumables per bed is reduced. The consumable cost of the department serves as a performance metric, which improves nurses’ sense of participation and self-discipline and effectively prevents the loss and waste of consumables.

## Limitations

At present, the management system has been studied in only one department, and it has been narrowly applied. Hence, the scope of this research remains to be expanded to other departments. The goal of consumable management is to bring all department consumables under one management system, but there has not been sufficient experience in coding the charges for high-value consumables in areas such as the operating room [30] and the intervention room. As this study evaluated a period from 2013 to 2019, some data might be missing because of the long time span, and the application of the systems was not evaluated comprehensively enough. When the matching of consumable collection and use was analysed in this study, relevant data from Group 1 were missing due to the long time span and hence could not be analysed.

## Conclusions

Our endocrinology department independently designed and developed an in**–**out storage management system based on computer information technology; this system could fully perform and track closed-loop management of all consumables of the department from application, storage inflow, collection, use, and fee deduction to storage outflow, verification, settlement, and statistical analysis. Cost control was effectively realized on the basis of ensuring the safe use of consumables [19]. The informational interconnection of the system facilitated not only the registration and in**–**out storage of the department’s consumable management but also the use and charging of supplies in the department. It standardized all aspects of the department’s consumable circulation; strengthened the supervision and the self-discipline of the personnel; and discouraged the emergence of unhealthy tendencies, theft, and corruption. Overall, this method of computer-assisted management led to scientific, institutionalized, standardized, and humanized consumable management in the department. Finally, the goal of reducing the “consumption proportion” was achieved, which ensured the scientific and rational application of medical consumables in clinical practice.

## Supplementary Information

**Additional file 1**. Satisfaction of warehouse keeper and collection staff. The dataset used to support the findings of this study are available from the corresponding author upon request.

## Data Availability

The data set used to support the findings of this study is available from the corresponding author upon request.

## Abbreviations

FMEA: Failure mode and effect analysis
HIS: Hospital information system
R&D: Research and development
RCA: Root cause analysis
SPD: Supply, processing, and distribution mode
LSD: Least significant difference
ICUs: Intensive care units

## References

[1] Liu T, Shen A, Hu X, Tong G, Gu W, Yang S (2016). SPD-based logistics management model of medical consumables in hospitals. Iran J Public Health.

[2] Coustasse A, Tomblin S, Slack C (2013). Impact of radio-frequency identification (RFID) technologies on the hospital supply chain: a literature review. Perspect Health Inf Manag.

[3] Zhu S (2012). The application of barcode technology in management of high value medical consumables. Zhongguo Yi Liao Qi Xie Za Zhi.

[4] Epstein RH, Dexter F (2000). Economic analysis of linking operating room scheduling and hospital material management information systems for just-in-time inventory control. Anesth Analg.

[5] Peine A, Hallawa A, Schöffski O, Dartmann G, Fazlic LB, Schmeink A (2019). A deep learning approach for managing medical consumable materials in intensive care units via convolutional neural networks: technical proof-of-concept study. JMIR Med Inform.

[6] Lehmann M, Philips M, Telesca C, Sariyar M, Holm J, Zetz E (2019). Components for material master data management in Swiss hospitals. Stud Health Technol Inform.

[7] Ahmadi E, Masel DT, Metcalf AY, Schuller K (2019). Inventory management of surgical supplies and sterile instruments in hospitals: a literature review. Health Syst (Basingstoke).

[8] Post A, Chappidi N, Gunda D, Deshpande N (2019). A method for EHR phenotype management in an i2b2 data warehouse. AMIA Jt Summits Transl Sci Proc.

[9] Davoody N, Koch S, Krakau I, Hägglund M (2019). Accessing and sharing health information for post-discharge stroke care through a national health information exchange platform—a case study. BMC Med Inform Decis Mak.

[10] Saeedi P, Petersohn I, Salpea P, Malanda B, Karuranga S, Unwin N, et al. Global and regional diabetes prevalence estimates for 2019 and projections for 2030 and 2045: Results from the International Diabetes Federation Diabetes Atlas, 9(th) edition. Diabetes Res Clin Pract. 2019;157:107843. 10.1016/j.diabres.2019.107843 31518657

[11] Tesfaye S, Boulton AJ, Dyck PJ, Freeman R, Horowitz M, Kempler P (2010). Diabetic neuropathies: update on definitions, diagnostic criteria, estimation of severity, and treatments. Diabetes Care.

[12] Senders JW (2004). FMEA and RCA: the mantras of modern risk management. Qual Saf Health Care.

[13] Alejo-Reyes A, Olivares-Benitez E, Mendoza A, Rodriguez A (2019). Inventory replenishment decision model for the supplier selection problem using metaheuristic algorithms. Math Biosci Eng.

[14] Williamson K, Lank PM, Cheema N, Hartman N, Lovell EO (2018). Comparing the Maslach Burnout inventory to other well-being instruments in emergency medicine residents. J Grad Med Educ.

[15] Kahlenberg CA, Nwachukwu BU, Schairer WW, McCormick F, Ranawat AS (2016). Patient satisfaction reporting for the treatment of femoroacetabular impingement. Arthroscopy.

[16] Beck EC, Nwachukwu BU, Mehta N, Jan K, Okoroha KR, Rasio J (2020). Defining meaningful functional improvement on the visual analog scale for satisfaction at 2 years after hip arthroscopy for femoroacetabular impingement syndrome. Arthroscopy.

[17] Kliemann N, Wardle J, Johnson F, Croker H (2016). Reliability and validity of a revised version of the General Nutrition Knowledge Questionnaire. Eur J Clin Nutr.

[18] Rachiotis G, Kourousis C, Kamilaraki M, Symvoulakis EK, Dounias G, Hadjichristodoulou C (2014). Medical supplies shortages and burnout among greek health care workers during economic crisis: a pilot study. Int J Med Sci.

[19] Liao HC, Chen MH, Wang YH (2014). The study of the optimal parameter settings in a hospital supply chain system in Taiwan. ScientificWorldJournal.

[20] Behzadifar M, Martini M, Behzadifar M, Bakhtiari A, Bragazzi NL (2020). The barriers to the full implementation of strategic purchasing and the role of health policy and decision-makers: past, current status, ethical aspects and future challenges. J Prev Med Hyg.

[21] Ford EW, Scanlon DP (2007). Promise and problems with supply chain management approaches to health care purchasing. Health Care Manage Rev.

[22] Kumar A, Cariappa MP, Marwaha V, Sharma M, Arora M (2016). Improving medical stores management through automation and effective communication. Med J Armed Forces India.

[23] Chen L, Suh BI (2017). Cytotoxicity and biocompatibility of resin-free and resin-modified direct pulp capping materials: a state-of-the-art review. Dent Mater J.

[24] Gorji HA, Mousavi S, Shojaei A, Keshavarzi A, Zare H (2018). The challenges of strategic purchasing of healthcare services in Iran Health Insurance Organization: a qualitative study. Electron Physician.

[25] Liu T, Shen A, Hu X, Tong G, Gu W (2017). The application of collaborative business intelligence technology in the hospital SPD logistics management model. Iran J Public Health.

[26] Yang C, Gu W, Liu T (2019). Application and evaluation of SPD based logistics management model for medical consumables in clinical nursing departments. Iran J Public Health.

[27] Wicks AM, Visich JK, Li S (2006). Radio frequency identification applications in hospital environments. Hosp Top.

[28] Woo BFY, Lee JXY, Tam WWS (2017). The impact of the advanced practice nursing role on quality of care, clinical outcomes, patient satisfaction, and cost in the emergency and critical care settings: a systematic review. Hum Resour Health.

[29] Awaya T, Ohtaki K, Yamada T, Yamamoto K, Miyoshi T, Itagaki Y (2005). Automation in drug inventory management saves personnel time and budget. Yakugaku Zasshi.

[30] Ito N, Chinzei M, Fujiwara H, Usui H, Hanaoka K, Saitoh E (2006). Outline and effectiveness of support system in the surgical center by supply, processing and distribution center (SPD). Masui.

