# Supplementary material for: Research, development, and evaluation of the practical effect of a storage inflow and outflow management system for consumables in the endocrinology department of a hospital
Source: BMC Med Inform Decis Mak. 2022 Jan 11;22:9. doi: 10.1186/s12911-021-01744-y (PMC8753857; doi:10.1186/s12911-021-01744-y)
Supplement: Supplementary file 1 — Additional file 1. Satisfaction of warehouse keeper and collection staff. The dataset used to support the findings of this study are available from the corresponding author upon request. [file 12911_2021_1744_MOESM1_ESM.pdf]

Satisfaction Survey

Department name:________________________ Date:________________________

Dear Sirs:

Thanks for your unremitting support for our Department about the Consumables delivery! in order to let us understand more about your requirements and advice :Please complete this Satisfaction Survey form to help us improve our selves !

Filling instructions: There were 10 items worth 10 points each, for a total of 100 points. The distance marked along the line corresponded to a satisfaction score from 0 to 10. please place a moving marker on the line to represent your satisfaction.

1. The time of collection:

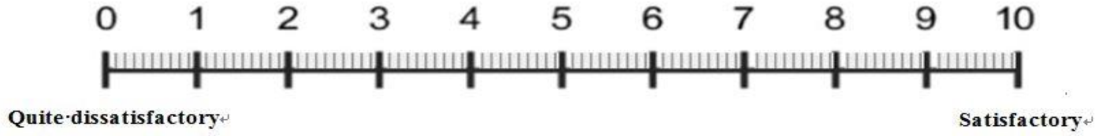

2. The duration of collection:

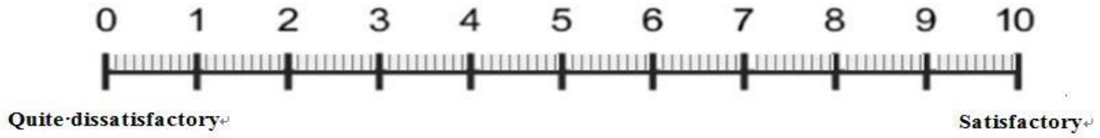

3. The accuracy of nurses' filling in the form:

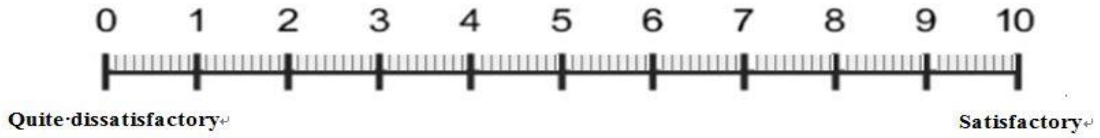

4. Planning of receiving:

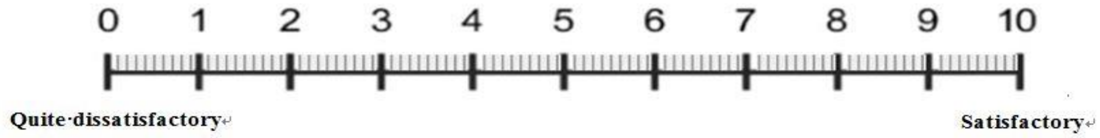

5. Times of replacement:

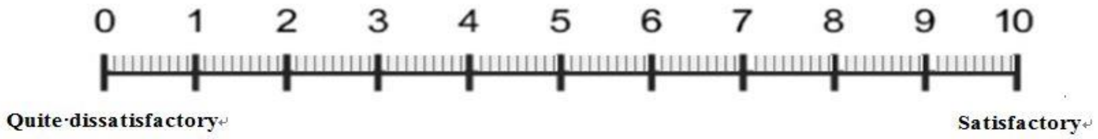

6. Dedicated management:

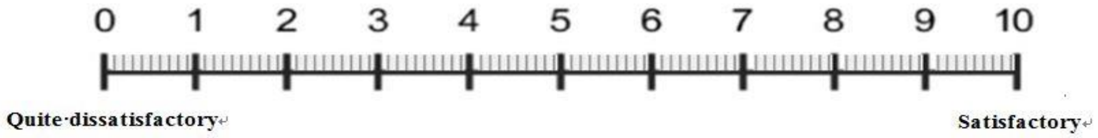

7. Reasonable process:

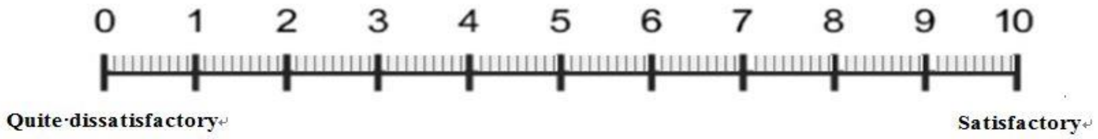

8. Time consumption:

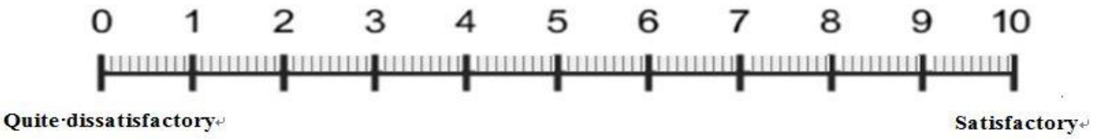

9. Response speed in case of abnormality:

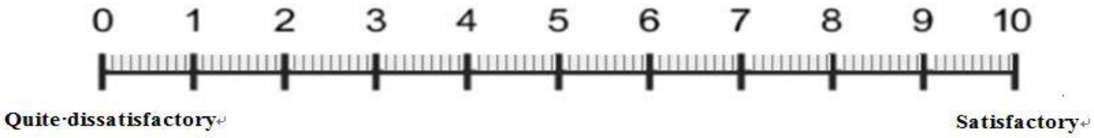

10. Overall evaluation:

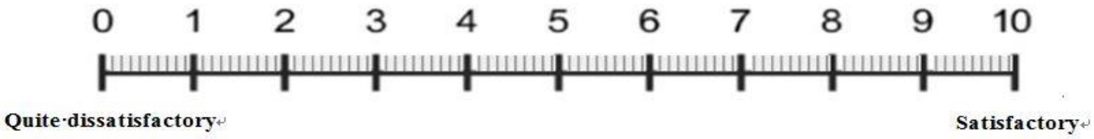

Total score:

☐Mare than 93 points (Satisfactory) :

☐Between 81 and 92 points (Relative satisfactory):

☐Between 50 and 80 points (Acceptable) :

☐Between 31 and 50 points (Dissatisfactory) ;

☐be1ow 30 points (Quite dissatisfactory)

Any complaints to department: ____

____

Amy advice to department:____

____
